# Novel Presentation of Cardiotoxicity and Other Complications in Tricyclic Antidepressant Poisoning

**DOI:** 10.7759/cureus.17181

**Published:** 2021-08-14

**Authors:** Hiep Nguyen, Ariel Kidron, Christopher Ghildyal, Shriya Veluri, Nghi Nguyen, Quan Nguyen, Hoang Nguyen

**Affiliations:** 1 Osteopathic Medicine, Nova Southeastern University Kiran C. Patel College of Osteopathic Medicine, Fort Lauderdale, USA; 2 Emergency Medicine, Nova Southeastern University Kiran C. Patel College of Osteopathic Medicine, Fort Lauderdale, USA; 3 Osteopathic Medicine, Nova Southeastern University Dr. Kiran C. Patel College of Osteopathic Medicine, Fort Lauderdale, USA; 4 School of Interdisciplinary Studies, University of Texas at Dallas, Richardson, USA; 5 Medicine, University of Saint Thomas, Houston, USA; 6 Clinical Sciences, Nova Southeastern University Dr. Kiran C. Patel College of Osteopathic Medicine, Clearwater, USA; 7 Basic Sciences, Nova Southeastern University Dr. Kiran C. Patel College of Osteopathic Medicine, Clearwater, USA

**Keywords:** psychiatric pharmacology, clinical psychiatry, cardiology, emergency medicine, emergency critical care, disaster medicine, neurology, critical care

## Abstract

Tricyclic antidepressant (TCA) is a known frequently used and highly potent antidepressant that serves as an unsuspecting source of acute human poisoning. We present a case of an Asian female in her mid-30s who suffered TCA toxidrome that manifested as severe cardiovascular toxicities including arrhythmia characterized by QT elongation that was managed emergently. Hemodynamics and ECG findings improved gradually following appropriate therapy in the intensive care unit. Following two days of treatment, the patient regained consciousness and after seven days the patient made a full clinical recovery and was discharged with no residual neurological effects. The relevant medical literature on TCA poisoning is reviewed.

## Introduction

Tricyclic Antidepressant (TCA) medications are prescribed as a psychiatric pharmaceutical agent in the treatment of depressive disorders. This is due to their potentially lethal toxicity and the difficulty of properly managing them. TCA overdoses have a 78.4% rate of hospitalization and a 0.73% fatality rate [1]. An overdose can potentially cause a multitude of anticholinergic effects that manifest through nervous, cardiovascular, and endocrine abnormalities, resulting in hypotension, loss of consciousness, and depressed respiratory rate, as well as dysrhythmias, which are the major cause of fatality. ECG is often utilized to stratify risk and guide treatment with sodium bicarbonate and activated charcoal. Herein, we present a case of how an intentional suicide attempt led to TCA toxicity, following an overdose, that was complicated by common organ toxicity, multiple cardiovascular events, and the lack of a proper management algorithm to aid physicians in improving treatment and patient outcomes. We specifically highlight the unique cardiac symptoms of QT interval prolongation and novel gastrointestinal findings and their treatment.

## Case presentation

**Figure 1:**
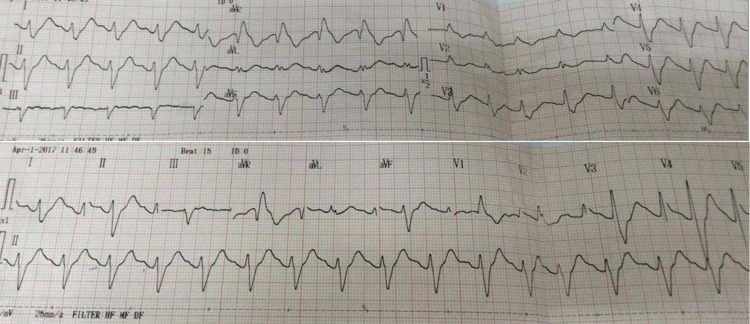
Electrocardiogram showing tachycardia and prolonged QRS complexes

A 35-year-old female patient presented to the emergency department at the American International Hospital of Vietnam with general convulsions, heart rate of 100 beats per minute, blood pressure at 70/40 mmHg, and a respiratory rate at 16 beats per min. She received sedation, intubation, mechanical ventilation, and vasopressors to raise her blood pressure. A rapid ECG (Fig. 1) revealed sinus tachycardia, prolonged QRS complexes, R waves >3mm, and R/S ratio >0.7 in augmented vector right (aVR). Laboratory study notably revealed a blood pH of 7.1 and bicarbonate (HCO3) of 13.8meq/L. Intravenous bicarbonate was administered and a gastroscopy revealed pills in the stomach with heavy mucosal congestion. After further discussion with family members, it was known that the medication ingested was three doses of a TCA drug known as amitriptyline.

The patient had multiple episodes of ventricular tachycardia (VT) and ventricular fibrillation (VF) and was defibrillated with continued 100mEq/kg of intravenous bicarbonate, repeated every minute until hemodynamic stabilized and QRS complex shortened. Also, 1.5mg/kg of lidocaine was administered and then referred to intensive care. In the ICU, the patient resumed vasopressors, bicarbonate, lidocaine and additionally was treated with electrolytes, proton pump inhibitor, activated charcoal, intravenous feeding, and mechanical ventilation via a bilevel positive airway pressure (BiPAP) machine.

After two days of treatment, the patient regained consciousness, the endotracheal tube was removed, BiPAP was continued, and bradycardia was noted for which the patient was given intravenous epinephrine. In the following days, the patient was removed from mechanical ventilation and discharged in stable condition. At the one-week follow-up, he made full recovery and was able to return to work. There were no signs of cardiovascular, endocrine, neurologic, or gastrointestinal complications. The patient was further referred to a psychiatrist for mental support.

## Discussion

TCA poisoning remains a leading cause of fatal drug overdose and results in higher rates of hospitalization than other drugs [2]. In addition to being rapidly absorbed in the gastrointestinal tract, TCAs are highly protein-bound and have a high volume of distribution, contributing to their long half-life, which can range from 31 to 46 hours. TCA acts in four pharmacokinetic manners that cause various toxic effects: inhibition of norepinephrine reuptake at nerve terminals, direct 𝛼-adrenergic blockade, anticholinergic actions, and quinidine-like effects on the myocardium [3].

Cardiotoxicity caused by TCA poisoning is indicated by the patient’s relatively specific ECG findings, including an enlarged QRS complex and sinus tachycardia. Prolongation of the QRS complex is attributable to the inhibition of sodium channels, causing the delayed propagation of depolarization and repolarization through the myocardium, while sinus tachycardia occurs due to inhibition of norepinephrine reuptake and anticholinergic action [3].

This patient had multiple episodes of VT and VF causing hemodynamic disturbances that were treated with defibrillation, continued intravenous sodium bicarbonate, lidocaine, and prolonged resuscitation. Intravenous sodium bicarbonate is the treatment of choice for cardiotoxicity as it narrows the QRS complex and resolves tachyarrhythmias and hypotension [4]. In patients with hemodynamic instability that is resistant to the above-mentioned interventions, intravenous lipid emulsion therapy may be considered. The patient was also hospitalized in a convulsive coma, which occurred in only 17% of patients in one retrospective study [5]. If the timeline of ingestion is established, gastric lavage may be performed to reduce absorption of TCA only when it can be delivered within one hour of ingestion and when the airway is intact. Single-dose activated charcoal may also be considered within one hour of ingestion; however multiple-dose activated charcoal is not recommended based on current literature [6].

Another unique presentation, in this case, was the heavy mucosal congestion revealed upon gastroscopy. While there is little to no literature regarding the mechanism of this side effect, one proposed explanation is that TCA blocks histamine H1 receptors, inhibiting histamine reuptake and stimulating mucus secretion and acid production in the stomach [7]. Combined with the anticholinergic effect of TCAs, which can reduce the muscle tone of the lower esophageal sphincters, larger amounts of histamine can increase the risk of gastroesophageal acid reflux [8].

It is important to note that in low- and middle-income countries, such as Vietnam, TCAs are still the primary treatment option for mental health conditions due to their low costs and lack of adequate drug regulation. Healthcare workers in these nations also tend to have minimal training in mental health and hence utilize a simple algorithm to prescribe medications without considering numerous individual patient factors

## Conclusions

This rare case of TCA poisoning with life-threatening sequelae could ultimately have led to a patient’s fatality; however, the physicians’ keen recognition and timely treatment prevented such an occurrence. It is the awareness of uncommon, yet important TCA toxicity symptoms that can prevent a delay in diagnosis. This report serves to further the current body of literature that exists on TCA toxicity and its rare but important associated symptom: cardiotoxicity. This patient’s QT prolongation and tachycardia along with the rest of his TCA overdose presentation underscore the value in further exploration of TCA pharmacological mechanisms in hopes to uncover and highlight similar serious complications. In particular, physicians practicing family medicine, emergency medicine, and neurology should be cognizant of these potential adverse anticholinergic effects of TCAs to deliver timely, appropriate care for their patients.
